# PKC-Mediated Modulation of Astrocyte SNAT3 Glutamine Transporter Function at Synapses *in Situ*

**DOI:** 10.3390/ijms19040924

**Published:** 2018-03-21

**Authors:** Wuxing Dong, Alison C. Todd, Angelika Bröer, Sarah R. Hulme, Stefan Bröer, Brian Billups

**Affiliations:** 1 Eccles Institute of Neuroscience, The John Curtin School of Medical Research, The Australian National University, 131 Garran Road, Canberra ACT 2601, Australia; dominicdongwuxing@gmail.com (W.D.); s1583068@sms.ed.ac.uk (A.C.T.); sarah.hulme@anu.edu.au (S.R.H.); 2 Centre for Discovery Brain Sciences, School of Biomedical Sciences, University of Edinburgh, Edinburgh EH8 9XD, UK; 3 Research School of Biology, The Australian National University, Linnaeus Way 134, Canberra ACT 2601, Australia; angelika.broer@anu.edu.au (A.B.); stefan.broeer@anu.edu.au (S.B.)

**Keywords:** *Slc38a3*, system N, protein kinase C, phorbol ester, protein trafficking, phosphorylation, biotinylation, glia, calyx of Held

## Abstract

Astrocytes are glial cells that have an intimate physical and functional association with synapses in the brain. One of their main roles is to recycle the neurotransmitters glutamate and gamma-aminobutyric acid (GABA), as a component of the glutamate/GABA-glutamine cycle. They perform this function by sequestering neurotransmitters and releasing glutamine via the neutral amino acid transporter SNAT3. In this way, astrocytes regulate the availability of neurotransmitters and subsequently influence synaptic function. Since many plasma membrane transporters are regulated by protein kinase C (PKC), the aim of this study was to understand how PKC influences SNAT3 glutamine transport in astrocytes located immediately adjacent to synapses. We studied SNAT3 transport by whole-cell patch-clamping and fluorescence pH imaging of single astrocytes in acutely isolated brainstem slices, adjacent to the calyx of the Held synapse. Activation of SNAT3-mediated glutamine transport in these astrocytes was reduced to 77 ± 6% when PKC was activated with phorbol 12-myristate 13-acetate (PMA). This effect was very rapid (within ~20 min) and eliminated by application of bisindolylmaleimide I (Bis I) or 7-hydroxystaurosporine (UCN-01), suggesting that activation of conventional isoforms of PKC reduces SNAT3 function. In addition, cell surface biotinylation experiments in these brain slices show that the amount of SNAT3 in the plasma membrane is reduced by a comparable amount (to 68 ± 5%) upon activation of PKC. This indicates a role for PKC in dynamically controlling the trafficking of SNAT3 transporters in astrocytes *in situ*. These data demonstrate that PKC rapidly regulates the astrocytic glutamine release mechanism, which would influence the glutamine availability for adjacent synapses and control levels of neurotransmission.

## 1. Introduction

Throughout the body, the neutral amino acid glutamine plays a central role in tissue nitrogen homeostasis and intercellular nutrition [1]. In the central nervous system, it is also critically involved in neurotransmitter recycling and metabolism. At synapses, the principal excitatory neurotransmitter glutamate is released to activate postsynaptic cells and is subsequently sequestered into neighbouring astrocytes to terminate the neurotransmitter signal [2]. In these astrocytes, glutamate is amidated to form glutamine, which is then transported out of astrocytes and back into neurons for hydrolysis back to glutamate, forming the glutamate–glutamine cycle (see reviews by [3,4,5,6]). This cycle is vital for maintaining the supply of presynaptic glutamate and sustaining synaptic communication [7,8,9,10,11]. Similarly, as the brain’s main inhibitory neurotransmitter GABA is synthesised from glutamate, glutamine shuttling from astrocytes to neurons is also important for the maintenance of inhibitory neurotransmission [12,13,14,15]. 

Release of glutamine by astrocytes is mediated by **S**odium-**N**eutral **A**mino Acid **T**ransporter SNAT3 (also known as SN1), the product of the gene *Slc38a3*, which is a member of the system N family of neutral amino acid transporters [16]. Amino acid transport by SNAT3 is coupled to the co-transport of one Na^+^ and the counter transport of an H^+^, resulting in an electroneutral transport process that is close to thermodynamic equilibrium under physiological conditions, and hence it can mediate transport of glutamine either into or out of cells [17,18,19]. Glutamine is the most abundant amino acid in the brain extracellular fluid and is therefore the major substrate for SNAT3 [20,21,22], although other neutral amino acids are also transported, including histidine and asparagine [19,23]. SNAT3 is found in a number of tissues, but predominantly in the liver, kidney and brain. Consequently, mice deficient in SNAT3 exhibit several characteristics including hypoglycaemia, ataxia, and growth restriction, which highlight its essential role in metabolic function [24]. In the brain, SNAT3 is localised to astrocytes surrounding synapses and is absent from neurons [25,26]. Due to its role in releasing glutamine that is used to maintain glutamatergic and GABAergic neurotransmission, the mechanisms which determine the cell surface expression of SNAT3 in astrocytes are of particular interest.

Dynamic trafficking of transporters in and out of the plasma membrane has been demonstrated for glutamate, GABA, glycine, dopamine, noradrenaline and serotonin transporters [27,28]. A common mechanism in controlling this trafficking is activation of protein kinase C (PKC) [29]. PKC exists in several isoforms that can be categorised as conventional, requiring diacylglycerol (DAG) and calcium for activation (PKCα, PKCβΙ, PKCβΙΙ and PKCγ); novel, which are activated by DAG but not calcium (PKCδ, PKCε, PKCη and PKCθ); and atypical, which are not activated by DAG or calcium (PKCζ and PKCι/λ) [30]. Astrocytes *in situ* express a number of these different PKC isoforms, including PKCα, PKCβΙ, PKCβΙΙ and PKCδ [31,32,33,34,35] and a variety of G-protein-coupled membrane receptors that can activate them [36]. The SNAT3 amino acid sequence contains a several consensus sequences for PKC phosphorylation [37,38], and previous studies in *Xenopus* oocytes and cultured cells have demonstrated that activation of PKC causes SNAT3 internalisation, possibly involving its phosphorylation [37,38,39,40]. However, expression of different PKC isoforms is tissue specific [41] and astrocytes express different isoforms than, for example, glioma cells or cultured glia at different stages of differentiation [42,43,44,45]. It is thus hard to infer the effects of PKC activation of SNAT3 *in vivo* from studies of cultured cells, and hence, the aim of this study is to investigate the effects of PKC activation on SNAT3 function and trafficking in astrocytes *in situ*, using acutely isolated brain slices. These astrocytes maintain their close relationship with pre- and postsynaptic neurons in an environment that closely mimics the true *in vivo* situation.

We have studied SNAT3 transporter function in astrocytes located immediately adjacent to the calyx of Held synapse in brain slices from the auditory brainstem of rats and mice. The calyx of Held is a large glutamatergic presynaptic terminal that can be visually identified in brain slices [46]. Astrocytes are in close association with this synapse [47] and heavily express SNAT3 [25]. This synapse is a pertinent model of neurotransmitter recycling because of its high neurotransmitter turnover [10]. We have previously shown that astrocytes adjacent to the calyx of Held play a central role in regulating neurotransmission by sequestering glutamate and releasing glutamine (via SNAT3) to maintain the presynaptic neurotransmitter supply [9,48,49]. Here, we show that activation of PKC rapidly reduces SNAT3 function at synapses by dynamic internalisation of transporters from the astrocytic plasma membrane, which will play an important role in regulating neurotransmitter supply in the central nervous system.

## 2. Results

### 2.1. Astrocytic SNAT3 Glutamine Transport in Acutely Isolated Brain Slices

**Figure 1:**
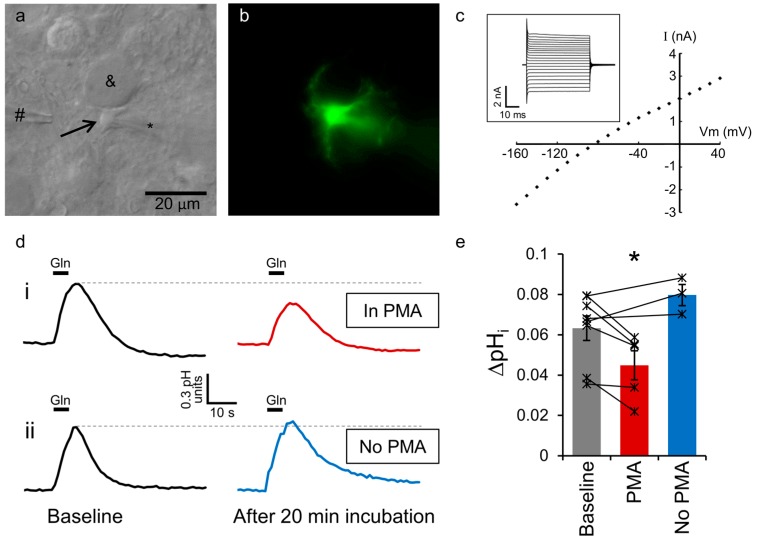
PKC activation reduces SNAT3 function. (**a**) Differential interference contrast image of a rat brainstem slice showing an astrocyte (arrow) adjacent to an MNTB principal neuron (&). The astrocyte is whole-cell voltage-clamped with a patch-pipette (*), and a puffer pipette (#) is visible near-by; (**b**) The same astrocyte visualised by excitation of the internal HPTS dye using 465 nm light. Astrocyte processes can be seen wrapping around the MNTB neuron; (**c**) Current responses in a voltage-clamped astrocyte to 50 ms voltage steps from a holding potential of −80 mV (inset). The current-voltage relationship is shown for 10 mV steps from −160 to +40 mV; (**d**) Example fluorescence recording of intracellular pH in a rat astrocyte as 10 mM glutamine is puff-applied for 5 s (black bars). In one cell (**d**) **i** initial baseline responses recorded for 10 min (black trace) are compared to responses after 20 min bath application of 400 nM PMA (red trace). A different cell (**d**) **ii** shows a consistent pH response at the beginning (black trace) and end of the recording (blue trace) when no PMA is added; (**e**) Averaged data from 5 rat cells show a glutamine induced alkalinisation of 0.058 ± 0.009 pH units (*n* = 5) at the beginning of recording, compared to 0.045 ± 0.007 pH units (*n* = 5) after 20 min PMA application (red bar; * *p* <0.01). Control data shows that incubation in PMA-free solution has no effect over the same time period (0.071 ± 0.004 pH units at baseline vs. 0.080 ± 0.005 pH units after incubation in artificial cerebrospinal fluid (aCSF); blue bar; *n* = 3). The values of each cell are shown by the individual points and represent an average of responses over 10 min recording in baseline, compared to an average of 10 min recording following 20 min of PMA or aCSF bath incubation.

To measure SNAT3 activity in individual brain astrocytes we examined astrocytes in brainstem slices from acutely isolated rat brains. Astrocytes immediately adjacent to principal neurons of the medial nucleus of the trapezoid body (MNTB) were whole-cell voltage-clamped and dialysed with the cell-impermeant fluorescent pH indicator HPTS (Figure 1a). Astrocytes were positively identified by the morphology visible under fluorescent illumination, showing a characteristic branching structure and close association with the calyx of Held synapse around the MNTB cell soma (Figure 1b). The cells also had electrical properties characteristic of astrocytes, showing no significant voltage activated currents over a range of voltage steps, a low membrane resistance and a resting membrane potential of around −80 mV (Figure 1c). SNAT3 glutamine transport was activated by pressure ejection of 10 mM glutamine from a puffer pipette placed 20–50 µm from the astrocyte soma (Figure 1a). This was performed in a cocktail of antagonists and ion channel inhibitors to prevent artefactual activation of glutamate receptors, GABA receptors, glycine receptors, sodium channels or potassium channels. As SNAT3 mediated glutamine transport is powered by the co-transport of Na^+^ and the counter transport of H^+^, it is electroneutral. However, it can be visualised by the intracellular alkalinisation observed upon glutamine application (Figure 1di), as we have previously demonstrated [9]. This response is positively identified as solely SNAT3-mediated by the affinity for glutamine, specificity for a range of amino acid substrates and lithium tolerance [9].

### 2.2. SNAT3 Function is Reduced by PKC Activation

Under control conditions, 5 s puff application of 10 mM glutamine, repeated every 2 min, results in a consistent SNAT3-mediated alkalinisation of 0.06 ± 0.01 pH units (*n* = 5). Following a period of baseline recording (>10 min), 400 nM PMA was added to the perfusing bath solution, to activate PKC. After 20 min of PMA application, the glutamine-induced alkalinisation in the same cells was reduced to 77 ± 6% of the original response (Figure 1di,e; *p* = 0.005 compared to baseline). In contrast, when no PMA was added to the bath, the degree of SNAT3 activation in each cell remained stable for the duration of the recording. Using the same time protocol as for PMA application, an average of 10 min of responses following incubation with control aCSF for 20 min was compared to the average of 10 min of recording prior to aCSF incubation. No significant change in SNAT3 activity was observed under these control conditions (Figure 1dii,e; 112 ± 5%; *n* = 3; *p* = 0.07), demonstrating the PMA-induced reduction of SNAT3 activity was not due to a time dependent run-down and likely represents the effect of PKC activation (*p* = 0.004 for the PMA effect compared to 20 min aCSF incubation).

### 2.3. Conventional PKC Isoforms Inhibit SNAT3 Function

**Figure 2:**
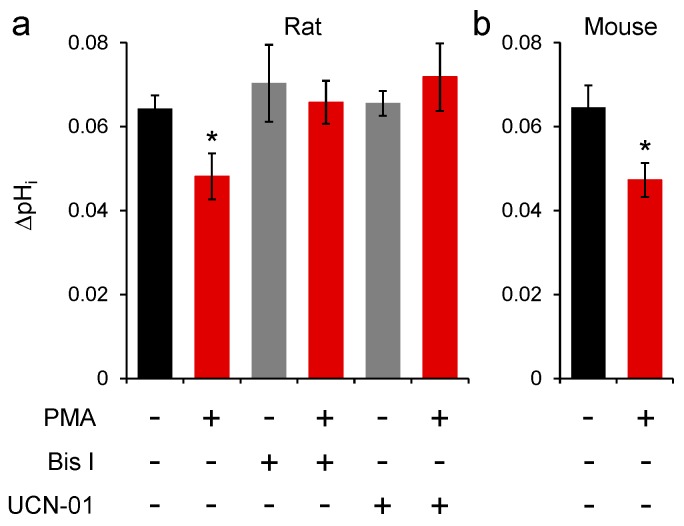
Conventional PKC isoforms reduce SNAT3 function. (**a**) Glutamine-induced alkalinisations in rat astrocytes, recorded after pre-incubation of slices in PMA or pharmacological analogues. 1 h incubation with 100 nM PMA reduces the SNAT3-meidated alkalinisation to 0.048 ± 0.005 pH units (*n* = 7; * *p* <0.05). 100 nM Bis I does not affect the SNAT3 function (0.070 ± 0.009 pH units; *n* = 7; *p* = 0.36) but does inhibit the action of PMA (0.066 ± 0.005 pH units; *n* = 7; *p* = 0.78). 10 nM UCN-01 also does not affect SNAT3 function (0.065 ± 0.003 pH units; *n* = 5; *p* = 0.86) but inhibits PMA action (0.071 ± 0.008 pH units; *n* = 5; *p* = 0.32); (**b**) Glutamine-induced alkalinisation in mouse astrocytes, similarly incubated for 1 h in control or 100 nM PMA containing solution. PMA induces a reduction in SNAT3-mediated alkalinisation to 0.047 ± 0.004 pH units (*n* = 13; * *p* <0.05).

Recording SNAT3 function in astrocytes for the long time periods used in Figure 1 was exceptionally difficult, and good statistical power was hard to achieve with this technique. Therefore, to confirm the effects of PKC activation on SNAT3 activity, and to investigate the PKC isoforms mediating the reduction of SNAT3 function, we incubated slices in PMA and pharmacological antagonists for 1 h before beginning recording. Under these conditions, comparing SNAT3 function between different cells in different brain slices, it was observed that PMA incubation induced an almost identical effect to applying PMA during recordings. Under control conditions the SNAT3-mediated alkalinisation was 0.064 ± 0.003 pH units (*n* = 24), which was reduced to 0.048 ± 0.005 pH units (to 75.0 ± 8.6%) by incubation in 100 nM PMA (Figure 2a; *n* = 7; *p* = 0.021). In the presence of bisindolylmaleimide I (Bis I) to inhibit PKC activation, the SNAT3 induced cellular alkalinisation was unchanged by incubation with PMA (Figure 2a; *p* = 0.80). Similarly, incubation with UCN-01, a selective inhibitor of conventional PKC isoforms [50], also prevented the effect of PMA on SNAT3 (Figure 2b; *p* = 0.32), suggesting that PKCα, PKCβΙ, PKCβΙΙ or PKCγ mediate inhibition of SNAT3.

### 2.4. PKC Inhibition of Mouse SNAT3 Function

All previous experiments we performed on rat brain slices, so to validate our results in mice we also performed a series of experiments in mouse astrocytes from the same brain area. The control SNAT3-induced alkalinisation was identical to that observed in rats, with 10 mM glutamine application inducing a pH change of 0.064 ± 0.005 pH units (*n* = 11). Similar to rats, incubation of mouse slices in PMA reduced the SNAT3-induced pH change to 0.047 ± 0.004 pH units (to 73.4 ± 6.3%; *n* = 13; Figure 2b; *p* = 0.017).

### 2.5. PKC Activation Induces Internalisation of SNAT3

**Figure 3:**
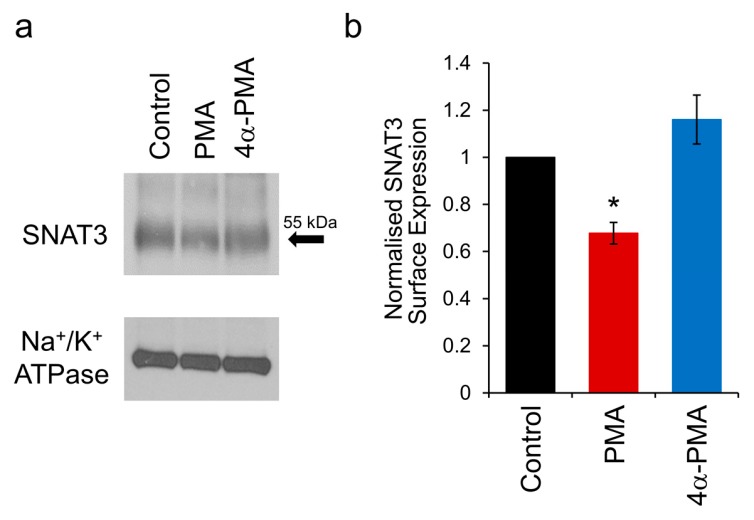
PKC activation internalises SNAT3. (**a**) A representative blot showing surface biotinylation of rat SNAT3 shown by western blot analysis. Slices were treated for 1 h in control, 100 nM PMA or 100 nM 4α-PMA prior to fixation and biotinylation. The SNAT3 surface expression is indicated by the band at 55 kDa (arrow, top panel). The gel loading control against the Na^+^/K^+^-ATPase is shown in the lower panel; (**b**) Quantification from 6 separate experiments shows a significant reduction of rat SNAT3 surface expression by incubation in 100 nM PMA (red bar; *n* = 6; * *p* <0.05), whereas the 4α-PMA control did not show an effect (blue bar; *n* = 4; *p* = 0.30).

The reduction in SNAT3 function by PMA could be due to the inhibition of transporter action or removal of transporters from the plasma membrane. Since PKC activation has been shown to induce the internalisation of a number of different classes of membrane transporters, we tested the hypothesis that activating PKC internalises SNAT3. We have previously demonstrated PKC induced internalisation of SNAT3 in *Xenopus* oocytes using biotinylation of surface proteins and immunohistochemistry [37]. We have applied the same technique here, using biotinylation of individual brain slices of rat MNTB, followed by detection of SNAT3 using western blotting with a SNAT3 antibody. One-hour pre-incubation of slices in 100 nM PMA resulted in a reduction in the surface expression of SNAT3 compared to control slices, which was not observed when slices were incubated with the inactive control compound 4α-PMA (Figure 3a). Quantification from repeat experiments showed that PKC activation with PMA induced a reduction of SNAT3 surface expression to 67.8 ± 4.6% of control (Figure 3b; *n* = 6; *p* = 0.014). In comparison, 4α-PMA had no effect on SNAT3 surface expression (Figure 3b; 116 ± 10%; *n* = 4; *p* = 0.30). These data demonstrate that PMA induces internalisation of SNAT3 from the astrocyte plasma membrane in acutely isolated brain slices.

## 3. Discussion

Our data show that the function of SNAT3 in astrocytes adjacent to synapses is rapidly downregulated by the activation of PKC. The degree of downregulation is mirrored by the proportion of transporters that are internalised upon PKC activation, indicating that the reduction in SNAT3 function can be entirely explained by a phosphorylation-mediated trafficking of the transporter from the plasma membrane.

We have used two separate approaches to investigate SNAT3 downregulation: recordings from individual cells in brain slices and measuring internalization in whole-tissue lysates. This combination greatly enhances our ability to draw firm conclusions from our data. For example, PKC activation by PMA may affect a variety of cellular processes, which could cause an artefactual change in the observed pH signal in Figure 1 and Figure 2, independent of effects on SNAT3 transport. The biotinylation experiment (Figure 3) allows us to discount this possibility, as this technique measures SNAT3 surface expression directly, and shows a clear internalization of the transporter. On the other hand, the whole-tissue lysates used in Figure 3 contain a number of different cell types, not just astrocytes. While SNAT3 in the brain is almost exclusively expressed by astrocytes, and is not observed in neurons or oligodendrocytes [19,25,26], some contamination of the signal by other cell types cannot be totally excluded. However, the single cell recordings (Figure 1 and Figure 2) show SNAT3 downregulation in positively identified individual astrocytes, providing reassurance that the internalization is astrocytic.

PKC-induced internalisation of SNAT3 has previously been clearly demonstrated by our group and others in *Xenopus* oocytes and cell lines [37,38]. However, studies of cultured astrocytes have revealed inconsistent results. Nissen-Meyer *et al.* demonstrate that SNAT3 in cultured rat astrocytes is phosphorylated by PKC [38], however Balkrishna *et al.* show that PKC activation does not affect SNAT3 function in astrocyte cultures [37]. In contrast to the rapid (~30 min) effects of PKC that we and others observe [37,38], Sidoryk-Wegrzynowicz *et al.* demonstrate an effect of PKC activation on SNAT3 internalisation in astrocyte cultures that takes 2–4 h [39]. These diverse effects observed in astrocyte cultures highlight the inadequacies of the culture model for understanding astrocytic function *in vivo*. For example, cultured astrocytes express significantly reduced amounts of SNAT3 compared to astrocytes *in vivo* [51], and the profile of PKC isoform expression is altered during differentiation of astrocytic cells [43,44,45]. These observations underscore the importance of our study on astrocytes *in situ* to understand the true physiological effects of PKC activation on SNAT3 function.

Our pharmacological data indicate that one of the conventional PKC isoforms mediates SNAT3 internalisation; PKCα, PKCβΙ, PKCβΙΙ or PKCγ. This is consistent with the *in vitro* phosphorylation of SNAT3 by PKCα or PKCγ [38], but indicates a different mechanism occurring in our experiments than the longer-term PKCδ-mediated effects on SNAT3 caused by manganese (II) exposure [39]. Whether direct phosphorylation of SNAT3 protein itself is required for internalisation is a controversial issue. Nissen-Meyer *et al.* show that *in vitro* SNAT3 is phosphorylated only at position S52, and that this is required for internalisation of SNAT3 when expressed in oocytes [38]. In contrast, Balkrishna *et al.* demonstrate that none of the PKC consensus sequences on the SNAT3 protein are required to be phosphorylated to observe a PKC-induced internalisation, and suggest that the target of PKC is elsewhere in the internalisation pathway [37]. In this study we have not directly investigated whether SNAT3 itself is phosphorylated. However, we have observed that both rat and mouse SNAT3 are down-regulated by PKC activation. This is significant because the serine residue present in the putative PKC phosphorylation site at positon 52 in rat is replaced by a proline residue in mouse and humans [38]. Thus, direct phosphorylation at position 52 cannot explain the PKC-mediated effects we observe and suggests that while PKC may possibly phosphorylate SNAT3, it could also affect transporter internalisation by phosphorylating other proteins in the trafficking pathway.

The mechanism of PKC-induced internalisation of SNAT3 has been shown to be caveolin dependent, but independent of dynamin [37]. It is also known that SNAT3 is a target for the ubiquitin ligase Nedd4-2 [39,52,53], which will tag the transporter for internalisation and degradation. As the interaction between Nedd4-2 and SNAT3 is enhanced by PKC activation [39], it is likely that the PKC-mediated internalisation that we observe involves this mechanism. 

PKC-mediated internalisation of a wide range of plasma membrane transporters has been reported including transporters for dopamine [54,55], noradrenaline [56,57], serotonin [58], glycine [59,60], GABA [61], neutral amino acids [62] and glutamate [63,64]. Ubiquitination is involved in the internalization of glycine transporters GlyT1 [65] and GlyT2 [66] and the glutamate transporter GLT-1 [67,68]. Specifically, Nedd4-2 mediated ubiquitination mediates the internalization of the dopamine transporter DAT [69] and GLT-1 [70,71,72], suggesting that common trafficking mechanisms exist between SNAT3 and a wide range of other amino acid and biogenic amine transporters. However, it is currently unknown how PKC influences the interaction between Nedd4-2 and any of these transporters.

What is the physiological function of PKC-mediated regulation of SNAT3? Astrocytes *in vivo* express a variety of Gq-coupled receptors that can activate PKC (reviewed by [36]). This includes receptors for glutamate (mGluR1 & 5), acetylcholine (M_1_), noradrenaline (α_1_), serotonin (5-HT_2A_), histamine (H_1_), ATP (P_2_Y), substance P, interleukin-1 beta [73] and neurosteroids (σ1 [74]). Hence, they are very sensitive to the neuronal activity around them and the subsequent intracellular signalling cascades can adjust the function of their plasma membrane transporters. Amino acid transporters have been shown to be present in intracellular vesicle pools, which are rapidly trafficked in and out of the plasma membrane via exo- and endocytosis mechanisms [7,75]. The control of these processes via Gq-coupled receptor signaling could represent a universal feedback mechanism to regulate cell function. In the case of excitatory synapse, SNAT3 and the astrocytic glutamate transporters (GLT-1 and GLAST) are both located in the same microdomain, adjacent to active synapses [25,76] where they are physically and functionally coupled [9,77]. They each play vital roles in controlling synaptic function, with glutamate transporters quickly removing glutamate to terminate the synaptic signal [78] and SNAT3 releasing glutamine for neuronal regeneration of glutamate [7,8,9]. PKC has similar effects on GLT-1 and SNAT3, causing down-regulation via internalisation with comparably rapid kinetics. Thus, astrocytic PKC signalling represents a central point that can dynamically control the glutamate-glutamine cycle, regulating the supply of neurotransmitter precursors and having a major influence on the efficacy of synaptic transmission. This negative feedback system, where activation of adjacent excitatory synapses would trigger suppression of further activity, could be tested experimentally and is an avenue for further study in this field.

## 4. Materials and Methods

### 4.1. Brain Slice Preparation

Wistar rats or C57BL/6 mice aged 10 to 15 days, of either sex, were killed by decapitation in accordance with the procedure approved by the Animal Experimentation Ethics Committee of the Australian National University (protocols A2014/59 approved 18 November 2014 and A2017/49 approved 21 November 2017). Brains were swiftly removed and transferred to oxygenated ice cold slicing solution containing (in mM): 2.5 KCl, 10 HEPES, 1.25 NaH_2_PO_4_, 10 glucose, 290 sucrose, 4 MgCl_2_, 0.1 CaCl_2_, and pH set to 7.3 with NaOH. Transverse brain slices, 100–140 µm thick, of the auditory brainstem containing the MNTB were made using an Integraslice 7550 PSDS tissue slicer (Campden Instruments, Loughborough, UK). Slices were placed in an incubation chamber at 37 °C for 30 min containing O_2_ bubbled aCSF composed of (in mM): 145 NaCl, 2.5 KCl, 10 HEPES, 1.25 NaH_2_PO_4_, 10 glucose, 1 MgCl_2_, 2 CaCl_2_, with osmolarity of 320 mmol·kg^−1^ and pH set to 7.3 with ~4.5 mM NaOH. Following incubation, the chamber and slices were left to rest in aCSF at room temperature and used within 8 h.

### 4.2. Electrophysiological Recording

Patch-clamp electrode and puffer pipettes were pulled from thick-walled borosilicate glass capillaries (GC150F-7.5; Harvard Apparatus, Holliston, MA, USA). Electrodes for astrocyte recordings had open tip resistance of 6–6.5 MΩ and were filled with internal solution containing (in mM): 130 KCl, 4 Glucose, 20 Sucrose, 10 HEPES, 0.1 EGTA, 0.025 CaCl_2_, 1.4 MgATP, 0.6 NaGTP, 0.25 8-Hydroxypyrene-1,3,6-trisulfonic acid (HPTS); pH set to 7.2 by KOH and osmolarity made up to 305 mmol·kg^−1^ by the addition of sucrose. During recording brain slices were continually perfused with aCSF (composition as above) at 32–34 °C, at a rate of 1 mL min^−1^, in a solution containing a cocktail of ion channel inhibitors (in µM): 40 DL-2-amino-5-phospohonopentanoic acid (APV), 10 dizocilpine maleate (MK801), 20 NBQX, 1 tetrodotoxin (TTX), 10 mM tetraethylammonium chloride (TEA), 1 strychnine and 10 bicuculline methochloride. Astrocytes were visualised with infrared differential interference contrast (DIC) optics and voltage-clamped at −80 mV using a HEKA EPC-10 double amplifier (HEKA Elektronik Dr. Schulze GmbH, Lambrecht/Pfalz, Germany), low-pass filtered at 10 and 2.9 kHz, and digitized at 25 kHz with Patchmaster software (HEKA). All chemicals were purchased from Sigma-Aldrich (Castle Hill, NSW, Australia), except APV, bicuculline, MK-801, NBQX (Abcam, Melbourne, VIC, Australia); and TTX (Latoxan, Portes-lès-Valence, France).

### 4.3. Fluorescent pH Imaging

Intracellular pH was fluorescently imaged by the inclusion of 250 µM HPTS in the internal solution. Cells were illuminated at 465 and 405 nm (100 ms exposure each) using a monochromator (Optoscan, Cairn Research, Faversham, UK). Images were collected, via a 505 nm dichroic mirror and 520 nm long-pass filter, with an electron-multiplying CCD camera (Cascade 512B, Photometrics, Tucson, AZ, USA) controlled by MetaFluor software (Molecular Devices, Sunnyvale, CA, USA). The frame rate was one pair of images per second, for a duration of 60 s per glutamine puff. SNAT3 was activated by a puff of 10 mM glutamine (dissolved in ASCF), pressure ejected from an adjacent pipette using a Picospritzer III (Parker Hannifin, Hollis, NH, USA). Intracellular pH was calculated from the ratio of fluorescence levels emitted at 465 and 405 nm after background subtraction [79]. Approximately calibration was performed by constructing a calibration curve using pipette solutions of known pH, which was linear in the range pH 6.8 to 8.6.

### 4.4. Surface Biotinylation

To determine plasma membrane expression of SNAT3, brain slices were first incubated for 1 h at 34 °C in control aCSF, PMA or 4α-PMA solution. Following this, they were washed 3 times with ice cold PBS at pH 8.0. Subsequently, slices were incubated for 30 min at room temperature in 0.5 mg/mL sulfo-NHS-LC-Biotin (Pierce, Rockford, IL, USA) in the continued presence of PMA or 4α-PMA. Slices were then lysed by incubation in lysis buffer (150 mM NaCl, 20 mM Tris·HCl, pH 7.5, 1% Triton X-100) for 30–60 min on ice. The lysate was centrifuged at top speed in a tabletop centrifuge for 15 min at 4 °C, and the supernatant was mixed with 50 µL of streptavidin-coated agarose particles (Pierce). The suspension was incubated at 4°C overnight with slight agitation. Agarose particles (approx. 50 µL) were washed 4 times with lysis buffer, and subsequently 20 µL 4-times concentrated sample buffer and 8 µL of 10-times concentrated reducing agent (Invitrogen, Scoresby, VIC, Australia) was added to the agarose particles to yield a sample volume of 80 µL. Samples were boiled for 5 min, and an aliquot of 30 µL was loaded on the gel.

To detect SNAT3 an affinity-purified custom antibody was used (Pineda Antibody Service, Berlin, Germany). The antiserum was generated in rabbits against protein-coupled peptide EIPRQTEMVELVPNGKHLE. This peptide sequence is unique to SNAT3/*Slc38a3* and shows no homology to other *Slc38* transporters, including other putative members of system N. The purified antibody was used at a dilution of 1:1000. It detects a band at 55 kDa, which can be blocked by the immunogenic peptide. The antibody was tested on HEK293 cells overexpressing rSNAT3/pcDNA3.1+ and resulted in a single 55 kDa band [80]. Antibody binding was detected by enhanced chemiluminescence using the ECL system according to manufacturer’s instructions using the provided secondary antibody at a dilution of 1:2000 (Amersham Pharmacia Biotech, Castle Hill, NSW, Australia). Bands on western blots were visualised by X-ray film exposure, with loading controls (Na^+^-K^+^-ATPase) visualised on the same blot after stripping and re-exposure. Band intensity was quantified using Image J (version 1.50, National Institutes of Health, Bethesda, MD, USA) and the intensities were normalised to the loading control.

### 4.5. Data Analysis

Results are presented as mean ± SEM. Stated n-numbers represent observations from individual brain slices. Statistical comparisons were performed using linear mixed effects (LME) ANOVA, implemented in R, and regarded as statistically significant if *p* <0.05.

## 5. Conclusions

Our results show that SNAT3 glutamine transporters in brain astrocytes are internalised by activation of PKC. This occurs in a physiological preparation, and within a brief timescale of a few minutes. As the role of this transporter at synapses is to mediate the shuttling of glutamine from astrocytes to presynaptic terminals for glutamate production, this dynamic regulation by PKC represents a potential mechanism for rapidly modulating the efficacy of synaptic communication.
